# The genome sequence of the European shag, *Gulosus* *aristotelis *(previously *Phalacrocorax aristotelis*) (Linnaeus, 1761)

**DOI:** 10.12688/wellcomeopenres.21119.2

**Published:** 2025-09-22

**Authors:** Hannah M. Ravenswater, Fiona Greco, Sarah J. Burthe, Emma J. A. Cunningham, Darwin Tree of Life Barcoding collective, Wellcome Sanger Institute Tree of Life Management, Samples and Laboratory team, Wellcome Sanger Institute Scientific Operations: Sequencing Operations, Wellcome Sanger Institute Tree of Life Core Informatics team, Tree of Life Core Informatics collective, Darwin Tree of Life Consortium

**Affiliations:** 1 The University of Edinburgh, Edinburgh, Scotland, UK; 2 UK Centre for Ecology & Hydrology, Wallingford, England, UK

**Keywords:** Gulosus aristotelis, Phalacrocorax aristotelis, European shag, genome sequence, chromosomal, Pelecaniformes

## Abstract

We present a genome assembly from an individual female *Gulosus aristotelis,* previously known as *Phalacrocorax aristotelis*, (the European shag; Chordata; Aves; Pelecaniformes; Phalacrocoracidae). The genome sequence is 1,279.1 megabases in length. Most of the assembly is scaffolded into 36 chromosomal pseudomolecules, including the Z and W sex chromosomes. Gene annotation of this assembly on Ensembl identified 16,474 protein coding genes. The mitochondrial genome has also been assembled and is 18.61 kilobases in length.

## Species taxonomy

Eukaryota; Opisthokonta; Metazoa; Eumetazoa; Bilateria; Deuterostomia; Chordata; Craniata; Vertebrata; Gnathostomata; Teleostomi; Euteleostomi; Sarcopterygii; Dipnotetrapodomorpha; Tetrapoda; Amniota; Sauropsida; Sauria; Archelosauria; Archosauria; Dinosauria; Saurischia; Theropoda; Coelurosauria; Aves; Neognathae; Suliformes; Phalacrocoracidae; *Phalacrocorax*; *Phalacrocorax aristotelis* (Linnaeus, 1761) (NCBI:txid126867).

Taxonomic note. We follow recent avian checklists (e.g. IOC, BirdLife/BOU) that place the European shag in *Gulosus aristotelis* (Linnaeus, 1761), reflecting molecular evidence separating the “shags” from *Phalacrocorax sensu stricto*. NCBI currently retains *Phalacrocorax aristotelis* (NCBI:txid126867); we treat this as a synonym and map our usage of *Gulosus* to the same taxon ID.

## Background

**Figure 1.**
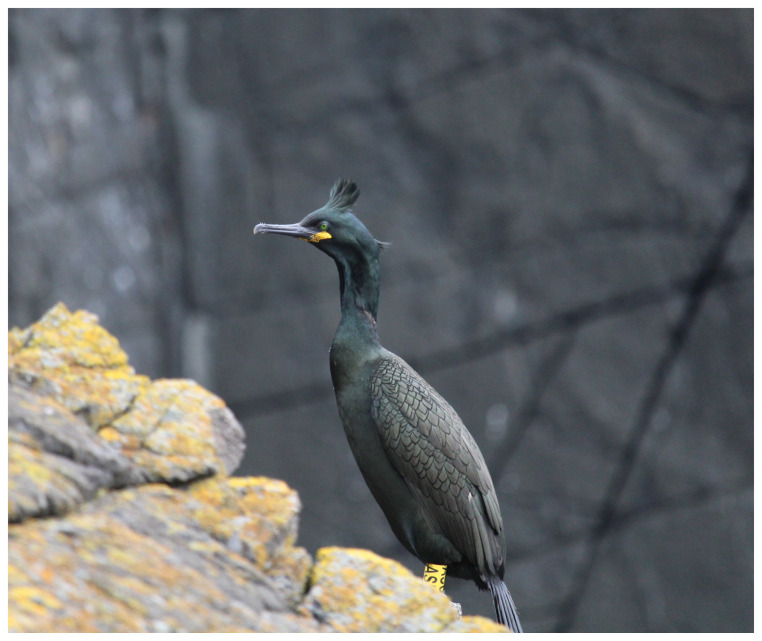
Photograph of a *Gulosus aristotelis* from the same study population as the specimen used for genome sequencing. Photograph by Fiona Greco.

Gulosus aristotelis (previously known as *Phalacrocorax aristotelis* and commonly known as the European shag), is a large seabird species within the cormorant family, Phalacrocoridae ( Croxall, 1987; Thanou *et al.*, 2017). European shags have dark-green plumage in adulthood ( Figure 1), but that can often appear black, a yellow gape, green eyes, black legs and feet and a crest during the breeding season ( Wanless & Harris, 1997). Juvenile plumage is lighter brown and present up to two years old ( Wanless & Harris, 1997). The species displays sexual dimorphism, with males weighing 1900 g on average, compared to 1600 g in females ( Daunt *et al.*, 2001). Sex is typically identified using vocalisations in adults ( Snow, 1960) and molecular techniques in juveniles ( Thanou *et al.*, 2013).

European shags are benthic foot-propelled pursuit divers, with a partially wettable plumage that facilitates diving in shallow waters but requires individuals to return to shore each day to dry and roost ( Grémillet *et al.*, 1998), increasing their vulnerability to inclement weather conditions. The species’ diet primarily consists of fish with some predation of crustations, with both temporal and spatial variability consumption of prey species ( Howells *et al.*, 2017; Howells *et al.*, 2018; Velando & Freire, 1999).

*Gulosus aristotelis* breeds colonially, creating nest sites on cliff ledges or cavities under rocks ( Velando & Freire, 2001; Wanless & Harris, 1997). European shags start breeding at 2 to 3 years ( Aebischer, 1986), with the lifespan of over 14 years on average ( Herborn *et al.*, 2014), with the oldest individual recorded on the Isle of May, an important seabird breeding colony where shags have been intensively monitored since the 1970s, at 23 years-old ( Hall, 2004). Breeding occurs seasonally, with shags laying on average a clutch of three eggs, which hatch asynchronously after an approximately 35-day incubation period ( Granroth-Wilding *et al.*, 2014; Snow, 1960). Both sexes provide parental care, in the form of incubation and provisioning until fledging of chicks at approximately 55 days ( Daunt *et al.*, 2007; Snow, 1960). During the non-breeding season, European shags remain coastal but may migrate variable distances from their breeding location. For example, on the Isle of May in Scotland, approximately 50% of the population migrates during the winter, with half the population remaining resident at the breeding area and half migrating up and down the entire East coast of the UK and, more rarely, across the North Sea to the Netherlands ( Acker *et al.*, 2023).

*Gulosus aristotelis* has a range that covers most of the coastline of Europe, with high concentrations the Atlantic coast and Mediterranean, and smaller populations at its southern range limit on the coast of North Africa ( GBIF Secretariat, 2023). With a European breeding population estimated at 152,000 ( BirdLife International, 2021), European shags are listed as ‘least concern’ but decreasing on the IUCN red list ( BirdLife International, 2018), with threats including climate change and extreme weather events, changing prey availability, pollution and disease ( BirdLife International, 2018; Hicks *et al.*, 2019). Similarly to other seabirds, European shags are ecologically important, not only in their role as marine predators but as key indicators of marine ecosystem health and environmental change ( Parsons *et al.*, 2008; Piatt & Sydeman, 2007).

 Long term monitoring programmes of seabird colonies, including European shags, such as that carried out on the Isle of May in Scotland (from which this specimen originates), are crucial in building a picture of the interacting processes that threaten seabirds. Therefore, the availability of this complete reference genome is a vital step in combining molecular tools with existing large life history datasets. For example, this genome will immediately aid the completion of an epigenetic clock, widening access to age data for birds that have not been individually marked as chicks, as well as the impact of stressors on biological aging where birds are of known age. This includes responses to infection and the genome sequence will be used to explore the molecular mechanisms of resistance and immunity against parasitism and disease, including long term chronic infections with parasites that are ubiquitous across individuals ( Granroth-Wilding *et al.*, 2017), and species differences in response to recent Avian Influenza outbreaks.

The genome of the European shag was sequenced as part of the Darwin Tree of Life Project and the Vertebrate Genomes Project (VGP). Here we present a chromosomally complete genome sequence for *G. aristotelis*, based on one female specimen from the Isle of May National Nature Reserve, Scotland.

## Genome sequence report

The genome was sequenced from a blood sample taken from one female *G. aritotelis* temporarily caught under licence from Isle of May National Nature Reserve, Scotland, UK (56.19, –2.57). A total of 42-fold coverage in Pacific Biosciences single-molecule HiFi long reads was generated. Primary assembly contigs were scaffolded with chromosome conformation Hi-C data. Manual assembly curation corrected 52 missing joins or mis-joins, reducing the scaffold number by 9.49%, and decreasing the scaffold N50 by 6.93%.

**Figure 2.**
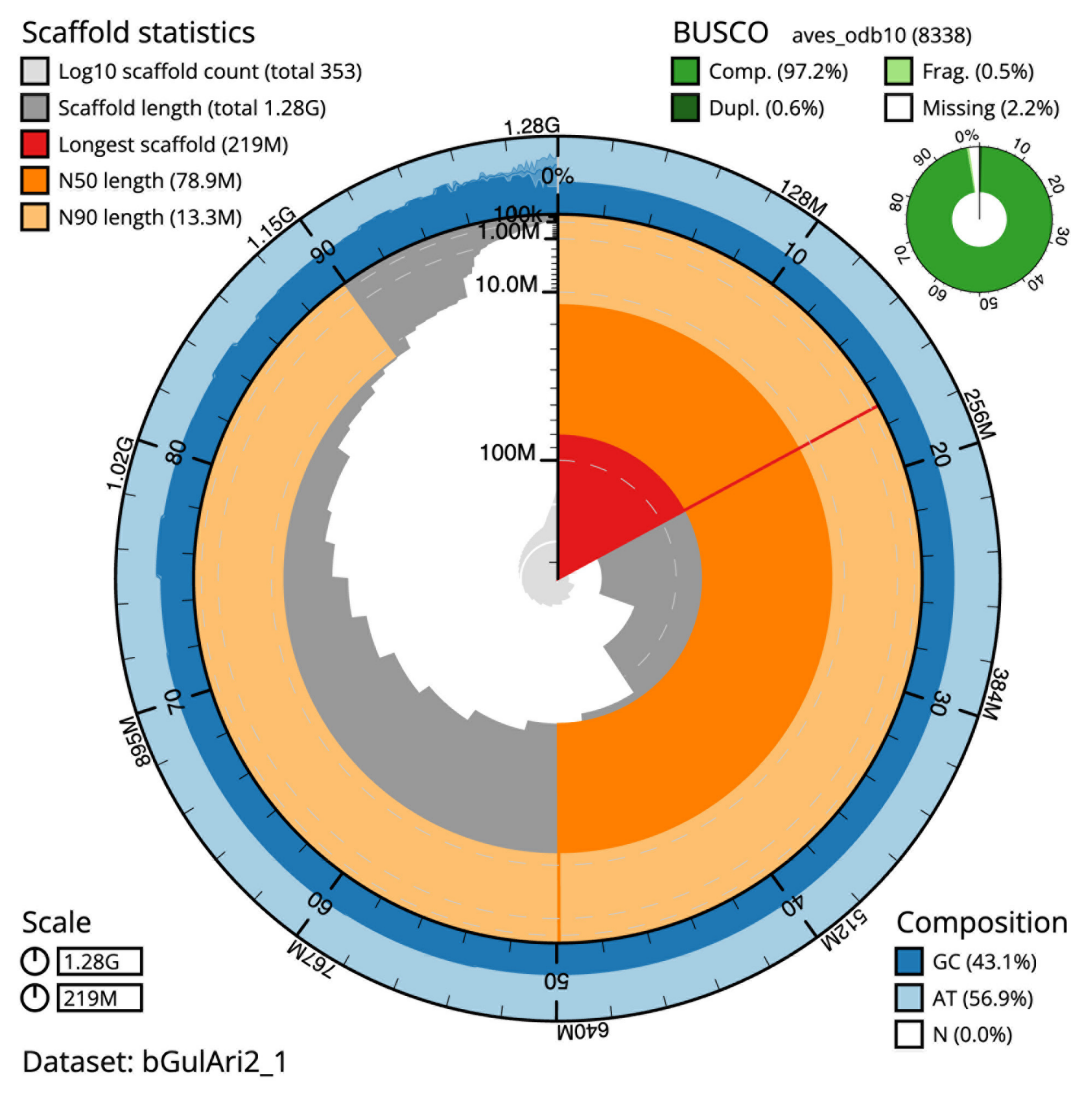
Genome assembly of *Gulosus aristotelis*, (previously *Phalacrocorax aristotelis)*, bGulAri2.1: metrics. The BlobToolKit snail plot shows N50 metrics and BUSCO gene completeness. The main plot is divided into 1,000 bins around the circumference with each bin representing 0.1% of the 1,279,134,750 bp assembly. The distribution of scaffold lengths is shown in dark grey with the plot radius scaled to the longest scaffold present in the assembly (219,240,020 bp, shown in red). Orange and pale-orange arcs show the N50 and N90 scaffold lengths (78,889,319 and 13,321,506 bp), respectively. The pale grey spiral shows the cumulative scaffold count on a log scale with white scale lines showing successive orders of magnitude. The outermost track of the plot shows the combined distribution of GC, AT and N percentages in the same bins as the inner plot, according to the key. A summary of complete, fragmented, duplicated and missing BUSCO genes in the aves_odb10 set is shown in the top right. An interactive version of this figure is available at https://blobtoolkit.genomehubs.org/view/bGulAri2_1/dataset/bGulAri2_1/snail.

**Figure 3.**
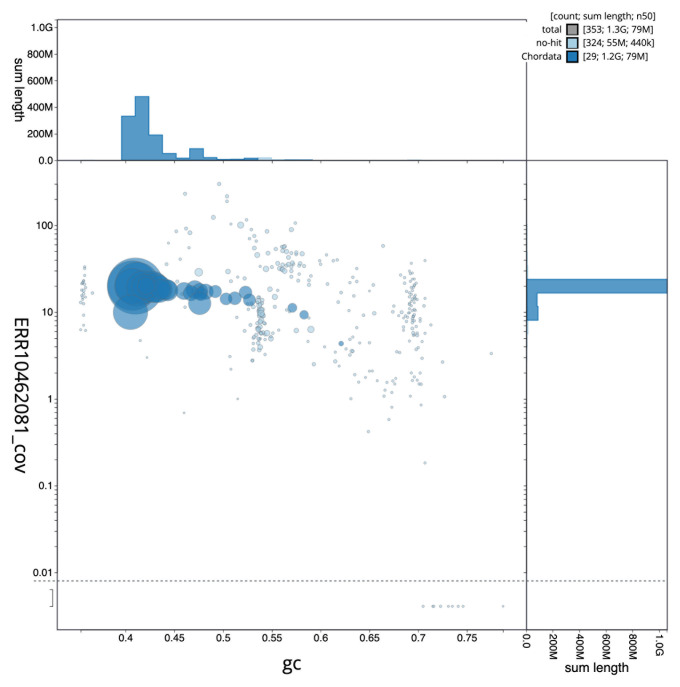
Genome assembly of *Gulosus aristotelis*, bGulAri2.1: BlobToolKit GC-coverage plot. Scaffolds are coloured by phylum. Circles are sized in proportion to scaffold length. Histograms show the distribution of scaffold length sum along each axis. An interactive version of this figure is available at https://blobtoolkit.genomehubs.org/view/bGulAri2_1/dataset/bGulAri2_1/blob.

**Figure 4.**
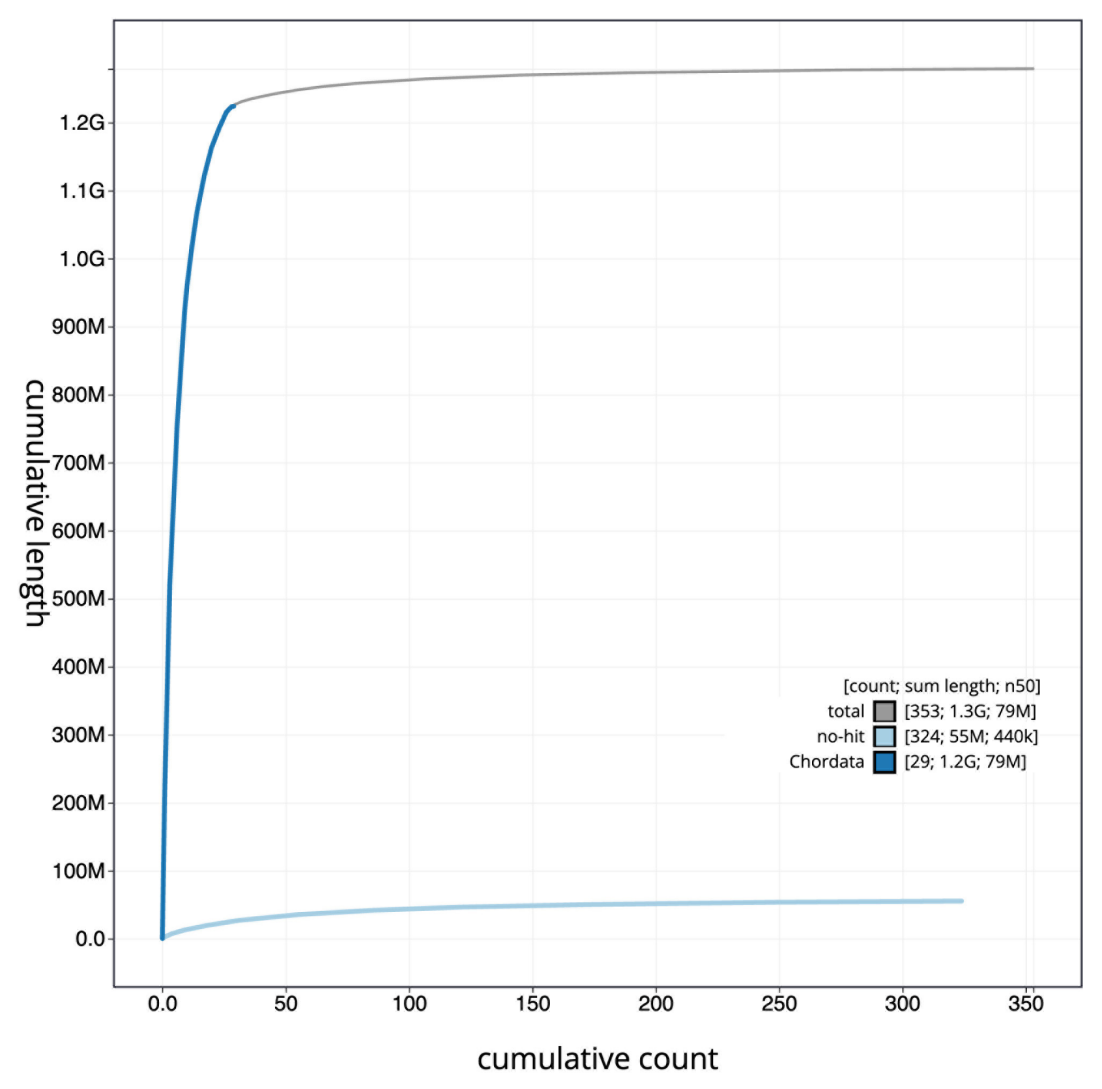
Genome assembly of *Gulosus aristotelis*, bGulAri2.1: BlobToolKit cumulative sequence plot. The grey line shows cumulative length for all scaffolds. Coloured lines show cumulative lengths of scaffolds assigned to each phylum using the buscogenes taxrule. An interactive version of this figure is available at https://blobtoolkit.genomehubs.org/view/bGulAri2_1/dataset/bGulAri2_1/cumulative.

**Figure 5.**
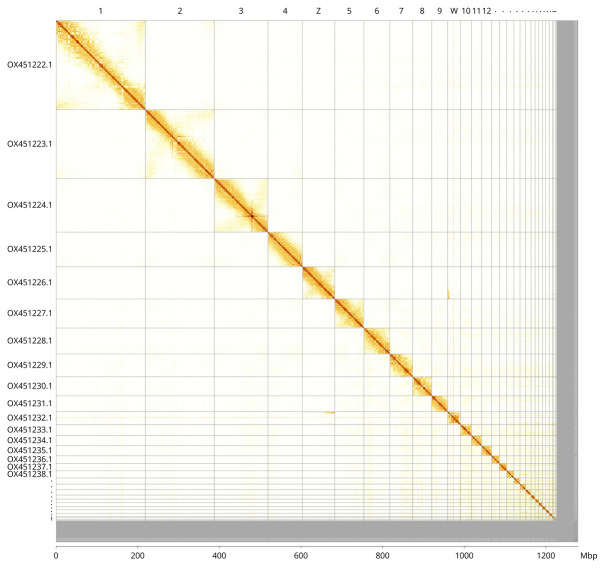
Genome assembly of *Gulosus aristotelis*, bGulAri2.1: Hi-C contact map of the bGulAri2.1 assembly, visualised using PretextView. Chromosomes are shown in order of size from left to right and top to bottom and are labelled along the axes with a megabase scale below. An interactive version of this figure in HiGlass may be viewed at https://genome-note-higlass.tol.sanger.ac.uk/l/?d=AXw7sVdmRrWp1DFeFKZT-Q.

The final assembly has a total length of 1,279.1 Mb in 352 sequence scaffolds with a scaffold N50 of 78.9 Mb ( Table 1). The snail plot in Figure 2 provides a summary of the assembly statistics, while the distribution of assembly scaffolds on GC proportion and coverage is shown in Figure 3. The cumulative assembly plot in Figure 4 shows curves for subsets of scaffolds assigned to different phyla. Most (96.21%) of the assembly sequence was assigned to 36 chromosomal-level scaffolds, representing 34 autosomes and the Z and W sex chromosomes. Chromosome-scale scaffolds confirmed by the Hi-C data are named in order of size ( Figure 5; Table 2). While not fully phased, the assembly deposited is of one haplotype. Contigs corresponding to the second haplotype have also been deposited. The mitochondrial genome was also assembled and can be found as a contig within the multifasta file of the genome submission.

**Table 1.** Genome data for *Phalacrocorax aristotelis*, bGulAri2.1.

Project accession data		
Assembly identifier	bGulAri2.1	
Species	*Gulosus aristotelis* (previously *Phalacrocorax aristotelis*)	
Specimen	bGulAri2	
NCBI taxonomy ID	126867	
BioProject	PRJEB57282	
BioSample ID	SAMEA10059652	
Isolate information	bGulAri2, female: blood sample (DNA, Hi-C and RNA sequencing)	
Assembly metrics *		*Benchmark*
Consensus quality (QV)	Primary: 61.7; alternate: 62.3; combined: 62.1	≥ 40
*k*-mer completeness	Primary: 97.25%; alternate: 82.08%; combined: 99.61%	*≥ 95%*
BUSCO **	C:97.2%[S:96.6%,D:0.6%],F:0.5%,M:2.2%,n:8,338	*S > 90%; D < 5%*
Percentage of assembly mapped to chromosomes	96.21%	*≥ 90%*
Sex chromosomes	ZW	*localised homologous pairs*
Organelles	Mitochondrial genome: 18.61 kb	*complete single alleles*
Raw data accessions		
PacificBiosciences SEQUEL II	ERR10462081, ERR10462082	
Hi-C Illumina	ERR10466815	
PolyA RNA-Seq Illumina	ERR11606292	
Genome assembly		
Assembly accession	GCA_949628215.1	
*Accession of alternate haplotype*	GCA_949628205.1	
Span (Mb)	1,279.1	
Number of contigs	575	
Contig N50 length (Mb)	11.4	
Number of scaffolds	352	
Scaffold N50 length (Mb)	78.9	
Longest scaffold (Mb)	219.24	
Genome annotation		
Number of protein-coding genes	16,474	
Number of non-coding genes	1,001	
Number of gene transcripts	26,595	

* Assembly metric benchmarks are adapted from column VGP-2020 of “Table 1: Proposed standards and metrics for defining genome assembly quality” from Rhie *et al*. (2021). ** BUSCO scores based on the aves_odb10 BUSCO set using version 5.3.2. C = complete [S = single copy, D = duplicated], F = fragmented, M = missing, n = number of orthologues in comparison. A full set of BUSCO scores is available at https://blobtoolkit.genomehubs.org/view/bGulAri2_1/dataset/bGulAri2_1/busco.

**Table 2.** Chromosomal pseudomolecules in the genome assembly of *Gulosus aristotelis*, bGulAri2.

INSDC accession	Chromosome	Length (Mb)	GC%
OX451222.1	1	219.24	41.0
OX451223.1	2	168.56	40.5
OX451224.1	3	131.34	41.0
OX451225.1	4	84.76	40.5
OX451227.1	5	71.72	42.0
OX451228.1	6	63.33	43.0
OX451229.1	7	55.81	43.0
OX451230.1	8	46.83	43.5
OX451231.1	9	39.05	42.0
OX451233.1	10	26.91	44.5
OX451234.1	11	24.85	44.0
OX451235.1	12	24.68	43.5
OX451236.1	13	19.06	47.0
OX451237.1	14	17.93	42.0
OX451238.1	15	17.37	46.0
OX451239.1	16	14.99	47.5
OX451240.1	17	13.73	48.0
OX451241.1	18	13.32	46.5
OX451242.1	19	9.84	47.5
OX451243.1	20	9.08	51.0
OX451244.1	21	8.89	52.5
OX451245.1	22	8.36	49.0
OX451246.1	23	8.19	52.5
OX451247.1	24	7.62	50.5
OX451248.1	25	3.89	57.0
OX451249.1	26	3.57	58.5
OX451250.1	27	2.68	47.5
OX451251.1	28	1.64	59.0
OX451252.1	29	1.11	54.5
OX451253.1	30	0.71	62.0
OX451254.1	31	0.63	57.5
OX451255.1	32	0.33	63.0
OX451256.1	33	0.23	61.5
OX451257.1	34	0.23	59.5
OX451232.1	W	31.35	47.5
OX451226.1	Z	78.89	40.5
OX451258.1	MT	0.02	44.5

The combined primary and alternate assemblies achieve an estimated QV of 62.1. The *k*-mer completeness is 97.25% for the primary assembly, 82.08% for the alternate haplotype, and 99.61% for the combined assemblies. The assembly has a BUSCO v5.3.2 completeness of 97.2% (single = 96.6%, duplicated = 0.6%), using the aves_odb10 reference set ( *n* = 8,338). Based on the Earth BioGenome Project Report on Assembly Standards ( September 2024), the EBP metric for the primary assembly is **7.C.Q61**, meeting the recommended reference standard.

Metadata for specimens, barcode results, spectra estimates, sequencing runs, contaminants and pre-curation assembly statistics are given at https://links.tol.sanger.ac.uk/species/126867.

## Genome annotation report

The *Gulosus aritotelis* genome assembly (GCA_949628215.1) was annotated at the European Bioinformatics Institute (EBI) using the Ensembl rapid annotation pipeline. The resulting annotation includes 26,595 transcribed mRNAs from 16,474 protein-coding and 1,001 non-coding genes ( Table 1; https://rapid.ensembl.org/Phalacrocorax_aristotelis_GCA_949628215.1/Info/Index).

## Methods

### Sample acquisition and nucleic acid extraction

A blood sample was taken from a female *Gulosus aristotelis* (specimen ID SAN0001768, ToLID bGulAri2) from the Isle of May National Nature Reserve, Scotland, UK (latitude 56.19, longitude –2.57) on 2021-06-29. The species was identified by Hannah Ravenswater (University of Edinburgh), Fiona Greco (University of Edinburgh) and Sarah Burthe (UK Centre for Ecology & Hydrology). Capture occurred via crook at the nest site during late chick rearing, and the individual released to the same location. Blood sampling was conducted by appropriately trained personal license holders, acting under a UK Home Office Project License in accordance with the Animals (Scientific Procedures) Act 1986. The blood sample was collected via brachial venepuncture of the live specimen using a 25-gauge needle. 75 μl blood was placed in 500 μl 95% ethanol, and then frozen at –80 °C within 120 mins.

The workflow for high molecular weight (HMW) DNA extraction at the Wellcome Sanger Institute (WSI) includes a sequence of core procedures: sample preparation; sample homogenisation, DNA extraction, fragmentation, and clean-up. In sample preparation, the bGulAri2 sample was weighed and dissected on dry ice ( Jay *et al.*, 2023). The blood sample was homogenised using a PowerMasher II tissue disruptor ( Denton *et al.*, 2023a).

HMW DNA was extracted using the Nanobind whole blood protocol ( Pacific Biosciences *et al.*, 2023). DNA was sheared into an average fragment size of 12–20 kb in a Megaruptor 3 system ( Bates *et al.*, 2023). Sheared DNA was purified by solid-phase reversible immobilisation ( Oatley *et al.*, 2023): in brief, the method employs a 1.8X ratio of AMPure PB beads to sample to eliminate shorter fragments and concentrate the DNA. The concentration of the sheared and purified DNA was assessed using a Nanodrop spectrophotometer and Qubit Fluorometer and Qubit dsDNA High Sensitivity Assay kit. Fragment size distribution was evaluated by running the sample on the FemtoPulse system.

RNA was extracted from a blood sample from bGulAri2 in the Tree of Life Laboratory at the WSI using the RNA Extraction: Automated MagMax™ *mir*Vana protocol ( do Amaral *et al.*, 2023). The RNA concentration was assessed using a Nanodrop spectrophotometer and a Qubit Fluorometer using the Qubit RNA Broad-Range Assay kit. Analysis of the integrity of the RNA was done using the Agilent RNA 6000 Pico Kit and Eukaryotic Total RNA assay.

Protocols developed by the WSI Tree of Life laboratory are publicly available on protocols.io ( Denton *et al.*, 2023b).

### Sequencing

Pacific Biosciences HiFi circular consensus DNA sequencing libraries were constructed according to the manufacturers’ instructions. Poly(A) RNA-Seq libraries were constructed using the NEB Ultra II RNA Library Prep kit. DNA and RNA sequencing was performed by the Scientific Operations core at the WSI on Pacific Biosciences SEQUEL II (HiFi) and Illumina NovaSeq 6000 (RNA-Seq) instruments. Hi-C data were also generated from a blood sample from bGulAri2 using the Arima2 kit and sequenced on the Illumina NovaSeq 6000 instrument.

### Genome assembly, curation and evaluation

Assembly was carried out with Hifiasm ( Cheng *et al.*, 2021) and haplotypic duplication was identified and removed with purge_dups ( Guan *et al.*, 2020). The assembly was then scaffolded with Hi-C data ( Rao *et al.*, 2014) using YaHS ( Zhou *et al.*, 2023). The assembly was checked for contamination and corrected using the gEVAL system ( Chow *et al.*, 2016) as described previously ( Howe *et al.*, 2021). Manual curation was performed using gEVAL, HiGlass ( Kerpedjiev *et al.*, 2018) and PretextView ( Harry, 2022). The mitochondrial genome was assembled using MitoHiFi ( Uliano-Silva *et al.*, 2023), which runs MitoFinder ( Allio *et al.*, 2020) and uses these annotations to select the final mitochondrial contig and to ensure the general quality of the sequence.

A Hi-C map for the final assembly was produced using bwa-mem2 ( Vasimuddin *et al.*, 2019) in the Cooler file format ( Abdennur & Mirny, 2020). To assess the assembly metrics, the *k*-mer completeness and QV consensus quality values were calculated in Merqury.FK ( Rhie *et al.*, 2020). The genome was analysed within the BlobToolKit environment ( Challis *et al.*, 2020) and BUSCO scores ( Manni *et al.*, 2021; Simão *et al.*, 2015) were calculated.

Table 3 contains a list of relevant software tool versions and sources.

**Table 3.** Software tools: versions and sources.

Software tool	Version	Source
BlobToolKit	4.1.7	https://github.com/blobtoolkit/blobtoolkit
BUSCO	5.3.2	https://gitlab.com/ezlab/busco
gEVAL	-	https://geval.org.uk/
Hifiasm	0.16.1	https://github.com/chhylp123/hifiasm
HiGlass	1.11.6	https://github.com/higlass/higlass
Merqury.FK	1.1.2	https://github.com/thegenemyers/MERQURY.FK
MitoHiFi	2	https://github.com/marcelauliano/MitoHiFi
PretextView	0.2	https://github.com/wtsi-hpag/PretextView
purge_dups	1.2.3	https://github.com/dfguan/purge_dups
YaHS	yahs-1.1.91eebc2	https://github.com/c-zhou/yahs

### Genome annotation

The Ensembl Genebuild annotation system ( Aken *et al.*, 2016) at the EBI was used to generate annotation for the *Gulosus aritotelis* assembly (GCA_949628215.1). Annotation was created primarily through alignment of transcriptomic data to the genome, with gap filling via protein-to-genome alignments of a select set of proteins from UniProt ( UniProt Consortium, 2019).

### Wellcome Sanger Institute – Legal and Governance

The materials that have contributed to this genome note have been supplied by a Darwin Tree of Life Partner. The submission of materials by a Darwin Tree of Life Partner is subject to the **‘Darwin Tree of Life Project Sampling Code of Practice’**, which can be found in full on the Darwin Tree of Life website here. By agreeing with and signing up to the Sampling Code of Practice, the Darwin Tree of Life Partner agrees they will meet the legal and ethical requirements and standards set out within this document in respect of all samples acquired for, and supplied to, the Darwin Tree of Life Project.

Further, the Wellcome Sanger Institute employs a process whereby due diligence is carried out proportionate to the nature of the materials themselves, and the circumstances under which they have been/are to be collected and provided for use. The purpose of this is to address and mitigate any potential legal and/or ethical implications of receipt and use of the materials as part of the research project, and to ensure that in doing so we align with best practice wherever possible. The overarching areas of consideration are:

•     Ethical review of provenance and sourcing of the material

•     Legality of collection, transfer and use (national and international) 

Each transfer of samples is further undertaken according to a Research Collaboration Agreement or Material Transfer Agreement entered into by the Darwin Tree of Life Partner, Genome Research Limited (operating as the Wellcome Sanger Institute), and in some circumstances other Darwin Tree of Life collaborators.

## Data Availability

European Nucleotide Archive: *Gulosus aristotelis* (European shag). Accession number PRJEB57282; https://identifiers.org/ena.embl/PRJEB57282 ( Wellcome Sanger Institute, 2023). The genome sequence is released openly for reuse. The *Gulosus aristotelis* genome sequencing initiative is part of the Darwin Tree of Life (DToL) project and the Vertebrate Genomes Project (VGP). All raw sequence data and the assembly have been deposited in INSDC databases. Raw data and assembly accession identifiers are reported in Table 1.

## Author information

Members of the Darwin Tree of Life Barcoding collective are listed here: https://doi.org/10.5281/zenodo.4893703.

Members of the Wellcome Sanger Institute Tree of Life Management, Samples and Laboratory team are listed here: https://doi.org/10.5281/zenodo.10066175.

Members of Wellcome Sanger Institute Scientific Operations: Sequencing Operations are listed here: https://doi.org/10.5281/zenodo.10043364.

Members of the Wellcome Sanger Institute Tree of Life Core Informatics team are listed here: https://doi.org/10.5281/zenodo.10066637.

Members of the Tree of Life Core Informatics collective are listed here: https://doi.org/10.5281/zenodo.5013541.

Members of the Darwin Tree of Life Consortium are listed here: https://doi.org/10.5281/zenodo.4783558.
