# Supplementary material for: The genome sequence of the European shag, Gulosus aristotelis (previously Phalacrocorax aristotelis) (Linnaeus, 1761)
Source: Wellcome Open Res. 2025 Sep 22;9:144. Originally published 2024 Mar 11. [Version 2] doi: 10.12688/wellcomeopenres.21119.2 (PMC12605584; doi:10.12688/wellcomeopenres.21119.2)
Supplement: Peer review history

# Reviewer report: Reviewer response for version 2

**DOI:** 10.21956/wellcomeopenres.27540.r137508

**Published:** 2025-11-11

**Authors:** Bengt Hansson

**Affiliations:** 1 Lund University, Lund, Sweden

This manuscript presents a high-quality genome assembly of the European shag ( *Gulosus aristotelis*). The assembly, derived from a female individual, spans ≈1.3 Mbp and includes 36 chromosomal pseudomolecules including both the Z and W chromosomes. The mitochondrial genome has also been assembled, and the genome contains an annotation (based on the Ensembl rapid annotation pipeline). I believe that this work contributes with valuable genomic resources for the species and will support and enable broader biodiversity and evolutionary studies. In general, I am happy with the paper, result and presentation, and find it to be a valuable addition to the field.

Are sufficient details of methods and materials provided to allow replication by others?

Yes

Is the rationale for creating the dataset(s) clearly described?

Yes

Are the datasets clearly presented in a useable and accessible format?

Yes

Are the protocols appropriate and is the work technically sound?

Yes

Reviewer Expertise:

Population genetics, evolutionary biology, avian genomics

I confirm that I have read this submission and believe that I have an appropriate level of expertise to confirm that it is of an acceptable scientific standard.

# Reviewer report: Reviewer response for version 1

**DOI:** 10.21956/wellcomeopenres.23361.r86201

**Published:** 2024-07-16

**Authors:** Xianzhao Kan

**Affiliations:** 1 Anhui Normal University, Wuhu, Anhui, China

The manuscript reports a genome assembly from an individual female, previously known as *Phalacrocorax aristotelis*, (the European shag; Chordata; Aves; Pelecaniformes; Phalacrocoracidae). The availability of this complete reference genome is a vital step in combining molecular tools with existing large life history datasets. Therefore, I think this is very meaningful and suitable for indexing in Wellcome Open Research. Some of my specific comments are below.

 Specific comments:

1. In the second paragraph of Background: “European Shags” should be “European shags”.
2. In the fourth paragraph of Background: “ *G.aristotelis*” should be “ *G. aristotelis*”.
3. In the first paragraph of Genome sequence report section: “The genome was sequenced from a blood sample taken from one female *Gulosus aritotelis* (previously *Phalacrocorax aristotelis*)…”, *Gulosus aritotelis* can be abbreviated to “ *G. aristotelis*”，and “previously *Phalacrocorax aristotelis*” can be removed as it is already covered in the Background section.
4. In Table 1, the size of mitochondrial genome in 18.61 kb, which is longer than typical avian mitogenomes. Please analyze the possible reasons for this.
5. In Table 2, the authors should further investigate the correlation between the chromosome length and GC content percentage, and analyze the possible reasons. Specifically, they should explain why the five chromosomes shorter than 1 Mb have a very high GC content percentage.
6. The figure legend for Figure 1, “Photograph of a *Gulosus aristotelis* (previously *Phalacrocorax aristotelis*) (bGulAri2) from same study…” should be changed to “Photograph of a *Gulosus aristotelis* (previously Phalacrocorax aristotelis) (bGulAri2) from the same study…”
7. The figure legend for Figure 2 should be modified to “Figure 2. Genome assembly of *Gulosus aristotelis* (previously Phalacrocorax aristotelis), bGulAri2.1: metrics.” Furthermore, “The blue and pale-blue area around the outside of the plot shows …” had better changed into “The blue and pale-blue areas around the outside of the plot show …”
8. In the first paragraph of Sample acquisition and nucleic acid extraction section: “The species was identified by Hannah Ravenswater (University of Edinburgh) and, Fiona Greco (University of Edinburgh) and Sarah Burthe (UK Centre for Ecology & Hydrology).” Should be “The species was identified by Hannah Ravenswater (University of Edinburgh), Fiona Greco (University of Edinburgh), and Sarah Burthe (UK Centre for Ecology & Hydrology).”

Are sufficient details of methods and materials provided to allow replication by others?

Yes

Is the rationale for creating the dataset(s) clearly described?

Yes

Are the datasets clearly presented in a useable and accessible format?

Yes

Are the protocols appropriate and is the work technically sound?

Yes

Reviewer Expertise:

Genomics, Bioinformatics

I confirm that I have read this submission and believe that I have an appropriate level of expertise to confirm that it is of an acceptable scientific standard, however I have significant reservations, as outlined above.

# Reviewer report: Reviewer response for version 1

**DOI:** 10.21956/wellcomeopenres.23361.r86203

**Published:** 2024-07-03

**Authors:** Nicolas J. Rawlence

**Affiliations:** 1 University of Otago, Dunedin, New Zealand

Ravenswater *et al.* have sequenced the nuclear and mitochondrial genome of the European shag *Gulosus aristotelis*, which will be a useful genomic resource for studying the evolution of cormorants and shags, including flight loss within the group. I hope my comments are helpful in revising this data note.

 The mitogenome that was produced is 18.61 kb in length. This suggests that there is a duplication within the mitogenome, probably of the Control Region/D-loop and surrounding genes (e.g. ND5, Cyt b) and tRNAs. It would be good to briefly comment on the increased size of the mitogenome and the reasons for it.

 The whole manuscript needs a thorough proof read as evidenced by the revisions noted below:

1. “Megabases in span”. Can you please change this to “megabases in size”.
2. Please make genus and species names in italics.
3. “European shags have dark-green plumage in adulthood but that can often appear black, a yellow gape, green eyes, black legs and feet and a crest during the breeding season ( Wanless & Harris, 1997)” should be “European shags have dark-green plumage in adulthood but can often appear black, a yellow gape, green eyes, black legs and feet, and a crest during the breeding season ( Wanless & Harris, 1997).” 
4. Please be consistent in use of “European Shag” vs “European shag”.
5. “both temporal and spatial variability consumption of prey species”. Something is missing here and needs rewording.
6. “an important seabird breeding colony where shags have been intensively monitored since the 1970s”. Put this section in parentheses, otherwise it is a very long sentence.
7. “For example, on the Isle of May in Scotland, approximately 50% of the population migrates during the winter, with half the population remaining resident at the breeding area and half migrating up and down the entire East coast of the UK and, more rarely, across the North Sea to the Netherlands ( Acker *et al.*, 2023)” I would mention Scotland in the first instance of Isle of May, not here or in other places latter in the text. “East coast” does not need a capital E.
8. “ *G.aristotelis* has a range that covers”. This is the start of a paragraph, so the genus species name needs to be spelled out in full.
9. “with high concentrations the Atlantic coast and Mediterranean”. There are some words missing here, please insert them.
10. “The genome was sequenced from a blood sample taken from one female *Gulosus aritotelis* (previously *Phalacrocorax aristotelis*)” Please make sure to spell the genus and species name correctly. There are many instances where this is not the case.
11. The figure three caption has the wrong genus.
12. “The species was identified by Hannah Ravenswater (University of Edinburgh) and, Fiona Greco (University of Edinburgh) and Sarah Burthe (UK Centre for Ecology & Hydrology)”. Please fix the grammar in this sentence.

Are sufficient details of methods and materials provided to allow replication by others?

Yes

Is the rationale for creating the dataset(s) clearly described?

Yes

Are the datasets clearly presented in a useable and accessible format?

Yes

Are the protocols appropriate and is the work technically sound?

Yes

Reviewer Expertise:

Evolutionary genetics of cormorants and shags

I confirm that I have read this submission and believe that I have an appropriate level of expertise to confirm that it is of an acceptable scientific standard, however I have significant reservations, as outlined above.
